# The potential of four legume trees for mercury phytoremediation and the role of arbuscular mycorrhizal fungi

**DOI:** 10.1007/s11356-026-38009-y

**Published:** 2026-07-11

**Authors:** Nadine Sommer, Yaqin Guo, Frank Rasche, Michael Helmut Hagemann, Christian Zörb

**Affiliations:** 1 Department Crop Physiology of Specialty Crops, Institute of Crop Science, University of Hohenheim, 70593 Stuttgart, Germany; 2 Department of Agronomy in the Tropics and Subtropics, Institute of Agricultural Sciences in the Tropics, University of Hohenheim, 70593 Stuttgart, Germany; 3 Department Quality of Plant Products, Institute of Crop Science, University of Hohenheim, 70593 Stuttgart, Germany; 4 International Institute of Tropical Agriculture, P.O. Box 30772-00100, Nairobi, Kenya; 5 Department of Pomology, Geisenheim University, Geisenheim, Germany

**Keywords:** Bioaccumulation, Gold mining, Heavy metal, Photosynthesis, Contamination, Reclamation, Ghana

## Abstract

Artisanal and small-scale gold mining (ASGM) in low- and middle-income countries often lacks adequate safety measures, leading to significant health risks and environmental mercury pollution. Phytoremediation, a plant-based method that utilizes plants to accumulate soil-borne contaminants such as heavy metals, has been verified to restore land for ecosystem services or even future farming. Therefore, this study evaluates the potential of four legume species typically found in Ghana, the world’s second largest gold exporter—*Acacia mangium*, *Gliricidia sepium*, *Leucaena leucocephala*, and *Senna siamea*—for the removal of mercury from contaminated soils, as well as potential trade-offs related to eco-physiological processes. It was further investigated whether arbuscular mycorrhizal fungi (AMF) inoculation could enhance mercury removal capacity. Predominantly, *A. mangium* consistently exhibited the highest mercury uptake and did not show signs of mercury toxicity. *G. sepium* showed moderate mercury uptake but suffered considerable physiological damage. *L. leucocephala* was resistant to mercury but accumulated only small amounts. *S. siamea* exhibited moderate mercury accumulation without physiological impairment. AMF inoculation did not significantly increase mercury uptake but appeared to mitigate physiological stress under mercury exposure. These results indicate that reforestation of abandoned gold mines with *A. mangium* may be a suitable starting point for phytoremediation of mercury and inoculation with AMF can provide additional protection against mercury toxicity.

**Supplementary Information:**

The online version contains supplementary material available at 10.1007/s11356-026-38009-y.

## Introduction

Artisanal and small-scale gold mining (ASGM) is the largest anthropogenic source of mercury pollution, which is caused by the amalgamation process used in gold extraction (Donkor et al. 2009; Boateng 2014). It was estimated that these mining activities emitted 838 tons of mercury to air in 2015, accounting for 38% of global anthropogenic mercury emissions (UN Environment  2019). Mercury amalgamation is the preferred method of almost all ASGM activities because it is simple and inexpensive (Donkor et al. 2009; Boateng et al. 2014). In addition to mercury contamination, gold mining drastically degrades soil health by removing the topsoil, which is rich in nutrients and organic matter (Aryee et al. 2003). Furthermore, the removal of vegetation has the effect of disrupting the flow of ecosystem services and matter fluxes, as well as increasing the risk of soil erosion (Schueler et al. 2011). What remains are “lunar” landscapes with rubbish dumps, abandoned excavations and vast, barren stretches of land (Aryee et al. 2003).

In Ghana, where gold mining is a predominant economic activity, the repercussions are particularly pronounced. In 2022, gold accounted for 48% of total exports in Ghana (OEC 2024). But mining, with its environmental impact, directly competes with agriculture, which was the largest employment sector for the Ghanaian people at 35.3% in 2022 (FAO 2024). The loss of farmland due to mining-related changes often forces farmers to move to new locations. As a result, farmers clear forests to create new farmland, indicating spillover effects of mining on neighboring areas (Schueler et al. 2011).

Contaminated soils can be remediated using chemical, physical or biological techniques. Chemical and physical treatments may irreversibly alter soil properties, destroy biodiversity, and are costly (Padmavathiamma and Li 2007). Since contaminated areas are extensive and funding is limited, a cost-effective biological soil remediation technique is needed to remove mercury without compromising soil fertility (Mertens et al. 2004). Phytoremediation, a process that uses plants to remove, transfer, stabilize or degrade contaminants in soil, is environmentally friendly and cost-effective (Hughes et al. 1997; Padmavathiamma and Li 2007). Such phytoremediation techniques include phytoextraction, phytostabilization, phytovolatilization, phytofiltration, and phytodegradation (Alkorta et al. 2004). Phytoextraction (also known as phytoaccumulation) is the uptake of contaminants from soil by plant roots and their translocation and accumulation in the aboveground biomass (Yoon et al. 2006). Translocation of metal ions into shoots is a crucial biochemical process and desirable for effective phytoextraction, as harvesting root biomass is difficult. Phytovolatilization describes a process whereby heavy metals are absorbed and transpired by plants (Yoon et al. 2006). Suitable plants for the phytoextraction of mercury should be tolerant to high mercury concentrations and should accumulate high concentrations of mercury in their harvestable above-ground tissues. Moreover, they should have a rapid growth rate with particularly high production of above-ground biomass, develop a well-branched root system, be adapted to the prevailing environmental and climatic conditions and should be easy to cultivate and harvest (Garbisu and Alkorta 2001; Ali et al. 2013).

Plants adapted to thrive in heavy metal-rich soils, known as ´metallophytes´, are categorized into metal excluders, metal indicators, and metal accumulators (Ali et al. 2013; Baker 1981). Metal excluders store heavy metals in their roots but restrict their transport to aerial parts, maintaining low metal concentrations in their shoots (Baker 1981). Metal indicators accumulate heavy metals in their aerial parts and generally reflect heavy metal concentrations in the substrate (Sheoran et al. 2011). Metal accumulators are preferred for phytoremediation since they gather high concentrations of metals in their above-ground biomass. This leads to efficient removal of contaminants from the environment (Baker 1981). The efficacy of metal accumulator plants can be assessed by the bioaccumulation factor (BAF) calculated as the heavy metal concentrations in plant shoots divided by the heavy metal concentrations in corresponding soils (Liu et al. 2020). Further, a high translocation factor (TF) calculated as heavy metal concentrations in plant shoots divided by the heavy metal concentrations in plant roots, is another key parameter (Xun et al. 2017). Ideal metal accumulators do not show high reduction in plant biomass when plants are grown in soils contaminated with heavy metals. This can be evaluated by the tolerance index (TI) calculated as the biomass of treated plants divided by the biomass of control plants (Diwan et al. 2010). Although over the past 20 years many scientists from different countries have investigated more than 200 plant species for their ability to accumulate and translocate mercury, no accumulator has been identified yet (Liu et al. 2020).

Generally, mercury is toxic for plants, it inhibits photosynthesis, nutrient uptake, nutrient transport affecting plant growth and biomass production (Lenti et al. 2002; Mei et al. 2021). To counteract those negative effects, plants may be inoculated with plant strengthening agents such as arbuscular mycorrhizal fungi AMF (Ferrol et al. 2016; Pirsarandib et al. 2022). Several studies have reported that certain AMF species, like *Glomus mosseae*, enhance the transport of heavy metals into the aboveground plant biomass (Weissenhorn et al. 1995; Singh et al. 2019) . However, other studies have shown that some AMF species, like *Funneliformis mosseae*, inhibit the translocation of heavy metals to the aboveground biomass (Shabani et al. 2016; Chamba et al. 2017; Salazar et al. 2018). This was explained by the observation that some heavy metals are bound to fungal hyphae, arbuscules, vesicles or vacuoles, which inhibits the transport of heavy metals to the plant tissue (Garg and Singh 2018; Motaharpoor et al. 2019).

The use of nitrogen-fixing legumes in the remediation of mercury-contaminated soils has the added benefit of increasing the biomass of subsequent plant species and thus the biomass production of the whole plant community (Frérot et al. 2006). The four legume tree species, *Acacia mangium* (Willd.) (synonym *Racosperma mangium* Willd.), *Gliricidia sepium* (Jacq.) Kunth, (synonym *Robinia sepium* Jacq.),* Leucaena leucocephala* (Lam.) de Wit and *Senna siamea* (Lam.) H.S.Irwin & Barneby (synonym *Cassia siamea* Lam.) were selected for their rapid growth and ability to establish and survive on degraded land in Ghana (Tetteh et al. 2015; Festin et al. 2019; Kusumaningtyas et al. 2021). Although none of the four species are native to Ghana, they are now widespread and commonly used throughout West Africa. *A. mangium*, native to Indonesia and northern Australia, was introduced around 1980 and is valued for its high-quality timber and use in land rehabilitation (Dupuy and Kanga 1990; Hegde et al. 2013). *G. sepium*, native to Mesoamerica, is widely planted for soil fertility improvement, agroforestry, and fuelwood (Osei-Tutu et al. 2018; Alamu et al. 2023). *L. leucocephala*, from Mexico and Central America, is used in Ghana as fodder, fuelwood, and for erosion control (Osei-Tutu et al. 2018; Zayed et al. 2018). *S. siamea*, native to tropical Asia, is introduced for soil remediation, agroforestry, shade, and timber, and has shown soil-improving effects in West African trials (Vanlauwe et al. 2005; Osei-Tutu et al. 2018).In addition, *A. mangium*, *G. sepium*, and *L. leucocephala* are nitrogen-fixing tree species, making them ideal for reforestation of degraded sites (Macedo et al. 2008; Tetteh et al. 2015).

While the majority of studies focus on the phytoextraction of heavy metals by grasses and herbs, this work is dedicated to tropical trees, which could potentially produce a higher biomass and consequently remove a larger amount of mercury (Liu et al. 2020). This study aims to investigate the phytoremediation potential of four tree species from Ghana in mercury-contaminated soils within gold mining areas. The optimal candidate should accumulate a high concentration of mercury in the above-ground biomass while remaining unharmed (Alkorta et al. 2004). Specifically, the objectives are as follows: (1) to assess the capacity of these species to accumulate mercury in their biomass, particularly in above-ground tissues, under varying levels of soil contamination; (2) to evaluate the physiological responses of these species to mercury exposure, including growth rates and photosynthesis; and (3) to explore the influence of AMF on the effectiveness of mercury uptake and resistance to mercury toxicity. Through this research, we identify and characterize species optimal for reforestation and ecological restoration of areas degraded by mercury pollution.

## Materials and methods

### Plant cultivation

Seeds of *S. siamea* and *L. leucocephala* were supplied by CSIR–Crops Research Institute (CSIR-CRI), Kumasi, Ghana. Seeds of *A. mangium* were purchased from Asklepios-Seeds (Bad Liebenzell, Germany), and seeds of *G. sepium* from Exotische Nutz- und Zierpflanzen (Seeheim-Jugenheim, Germany). To prepare the seedlings, the dormancy of the seeds of *A. mangium* (Am), *L. leucocephala* (Ll) and *S. siamea* (Ss) was broken by scarification of the seed coat, acid, or hot water treatment. *G. sepium* (Gs) did not require a pretreatment to break seed dormancy. After pretreatment, seeds were soaked in water for 24 h. Seeds were placed in germination trays and exposed to a 12/12 h light/dark cycle at temperatures of 25/20 °C in a climate chamber for 11 days. Subsequently, seedlings were transplanted into planting trays and placed in a greenhouse. A mixture of sand and vermiculite (5:3 by volume) was used as substrate. Prior to use, sand and vermiculite were autoclaved at 120 °C for 15 min and then completely dried at 80 °C. Greenhouse conditions were maintained at 26 °C with an average relative humidity of 56%. To provide artificial illumination, high-pressure sodium lamps were utilized (SONT Agro 400 W; Philips, Amsterdam, Netherlands) with a photoperiod of 12 h (PAR, 210—270 µmol m^−2^ s^−1^).

### Arbuscular mycorrhizal fungi (AMF)

The AMF strain *Rhizophagus irregularis* Błaszk., Wubet, Renker & Buscot, 2009 QS81 was supplied by INOQ GmbH (Schnega, Germany). The inoculum of *R. irregularis* was prepared from arbuscular mycorrhizal root fragments of *Trifolium pratense* grown in sand: vermiculite (35:65 by volume). The inoculum contained 100 million propagules per kg of powder (as vesicles and spores according to INOQ GmbH). Photographs of the experimental setup are provided as supplementary figure S1–S2.

### Experimental design

Two consecutive greenhouse experiments were carried out to avoid too many factors in one design. The greenhouse conditions were as described above. In the first experiment, the effect of a lower (12.5 mg kg^−1^) and a higher (25.0 mg kg^−1^) mercury concentration in the substrate on the tree species was investigated. These concentrations were selected based on the results of previous experiments (not reported here), which showed that they elicited physiological responses without causing severe damage to the plants. Higher concentrations caused mortality, while lower concentrations had little effect. This experiment is referred to as the ‘mercury concentration experiment’. Based on the results of the ‘mercury concentration experiment’ experiment, the higher mercury concentration was chosen for the second experiment, in which AMF was grown with only one mercury concentration. This experiment is referred to as the "mercury-AMF experiment".

#### Mercury-concentration experiment

Seedlings were transplanted into their final pots 11 weeks after germination pretreatment. To prevent mercury leaching from drainage water, 1 L plastic buckets without holes in the bottom were used. The substrate was a mixture of sand and vermiculite (19:1 by weight) with the addition of 1 g kg^−1^ controlled-release fertilizer (Osmocote® Exact Mini 3–4 M, NPK 15:3.9:9.1 + 1.2 Mg + trace elements, ICL, Netherlands). Prior to use, sand and vermiculite were autoclaved at 120 °C for 15 min and then completely dried at 80 °C. A randomized block design with five blocks was applied in a two-factorial design including tree species (*A. mangium*, *G. sepium*, *S. siamea*, and *L. leucocephala*) and mercury concentration (0, 12.5, and 25 mg Hg kg⁻^1^). Due to poor germination, *L. leucocephala* was only represented in three blocks, resulting in three replicates per treatment for this species and five replicates for the others. Mercury was applied in the form of a mercury chloride (HgCl_2_) solution, with the solution volume adjusted to achieve a final mercury concentration in the substrate of either 12.5 mg kg^−1^ or 25 mg kg^−1^14 weeks after germination. Eight weeks after mercury treatment, plants were harvested and separated into roots, stems, and leaves. Roots were shortly washed three times in deionized water. Plant samples were air dried at room temperature for dry weight determination.

#### Mercury-AMF experiment

Six weeks after germination, plants were divided into control (x) and AMF-inoculated (AMF) groups and transplanted into 1.0 L pots with 750 g of substrate. The substrate was a mixture of sand and vermiculite (19:1 by weight) with the addition of 0.5 g kg^−1^ controlled-release fertilizer (Osmocote® Exact Mini 3–4 M, NPK 15:3.9:9.1 + 1.2 Mg + trace elements, ICL, Netherlands). In the mercury–AMF experiment, a lower concentration of controlled-release fertilizer was applied than in the mercury-concentration experiment, following the recommendation of INOQ GmbH to enable AMF colonization. For the AMF-treated plants, the substrate was mixed with 0.075 g AMF inoculum (a ratio of 0.1 g kg^−1^). A randomized complete block design with four blocks was applied in a three-factorial design including tree species (four species), mercury concentration (0 and 25 mg Hg kg⁻^1^), and AMF inoculation (presence or absence), resulting in four replicates per treatment. Twelve weeks after germination 5 mg Hg kg^−1^ (3.75 mg pot^−1^) was applied as a mercuric chloride (HgCl_2_) solution every week for a period of 5 weeks to reach a final concentration of 25 mg Hg kg^−1^. Six weeks after the first mercury application, the plants were harvested as described in the mercury-concentration experiment. Root samples from each plant were divided into two subsamples: one was stored at − 80 °C to determine AMF colonization, and the other was used for mercury concentration analysis. For this, roots were cut into 1 cm lengths and cleared with 10% NaOH in a 70 °C water bath for 45 min and then soaked in 1% HCl for 1 min at room temperature. Roots were stained with 2% Parker Quink blue ink (Yon et al. 2015) in a 70 °C water bath for 30 min. The roots were rinsed with tap water and then stored in a lactoglycerol solution (lactic acid, glycerol, H_2_O in a ratio of 1:1:1). Thirty fragments from each plant were randomly selected, placed on a microscope slide, and examined under a light microscope for mycorrhizal colonization (Trouvelot et al. 1986).

### Mercury concentration

Plant samples were ground and homogenized using a mill with zirconium oxide grinding balls (MM40, Retsch GmbH, Haan, Germany). Subsequently, 0.2 g of the homogenized material was moistened with 1 mL of deionized H_2_O and digested in 2.5 mL of 69% HNO_3_ in a microwave-heated UltraCLAVE III digestion unit (MLS-MWS GmbH, Leutkirch, Germany). One mL of the microwave pressure digestion solution was added to 1 mL of dilution solution (2% cysteine, 7% isopropanol made up to 1000 mL with 1% HNO_3_) and 0.1 mL of 50 ppb rhodium standard solution and then made up to 10 mL with ddH_2_O. The mercury concentration was analyzed using a NexION 300 × inductively coupled plasma mass spectrometer (PerkinElmer LAS GmbH, Rodgau, Germany).

#### Gas exchange and biomass determination

All plants were analyzed weekly for their stem height using a scale and defined as the height from the substrate surface to the top of the main shoot (photographs in supplementary figure S3).

The gas exchange (CO_2_ and H_2_O) of the youngest fully developed leaf was determined weekly (GFS-3000, Heinz Walz GmbH; 3010-S standard measuring head with 3040-L LED light source, head area of 2.5 cm^2^). The area of leaves that did not fill the measuring head was determined using ImageJ.

The tolerance index (TI) was calculated in this work using the following equations (Diwan et al. 2010). TI values above 1 indicate a net increase in biomass and imply that the plants have developed heavy metal tolerance, while TI values below 1 indicate a net decrease in biomass and a stressed state of the plants.

$$TI= \frac{\mathrm{Biomass}\;\mathrm{of}\;\mathrm{treated}\;\mathrm{plants}\;(g\;{\mathrm{plant}}^{-1})}{\mathrm{Biomass}\;\mathrm{of}\;\mathrm{control}\;\mathrm{plants}\;(g\;{\mathrm{plant}}^{-1})}$$

The bioaccumulation factor (BAF) was calculated using the following equation (Liu et al. 2020). The initial Hg concentration in the substrate (12.5 or 25.0 mg Hg kg^−1^) was used to calculate the BAF.

$$BAF= \frac{Hg\;\mathrm{concentration}\;\mathrm{in}\;\mathrm{plant}\;\mathrm{shoot}}{Hg\;\mathrm{concentration}\;\mathrm{in}\;\mathrm{substrate}}$$

The translocation factor (TF), i.e., the ability of a plant to translocate metal ions from the roots to the shoots, was calculated as follows:

$$TF=\frac{Hg\;\mathrm{concentration}\;\mathrm{in}\;\mathrm{plant}\;\mathrm{shoot}}{Hg\;\mathrm{concentration}\;\mathrm{in}\;\mathrm{plant}\;\mathrm{root}}$$

### Statistical analysis

Mercury concentration, assimilation rate, stem height, biomass, TI, BAF, TF, and AMF were analyzed using ANOVA with SAS software 9.4 (SAS Institute, Cary NC, USA) with proc mixed. Least squares means were calculated for comparison between control and treated plants. The significance level was set at *p* ≤ 0.05. Model assumptions such as normality, homoscedasticity, and independence were verified, when necessary, log-transformation was applied, and post hoc comparisons were conducted using the least significant difference (LSD) test to ensure accurate interpretation of the differences.

## Results

### Mercury accumulation

**Fig. 1:**
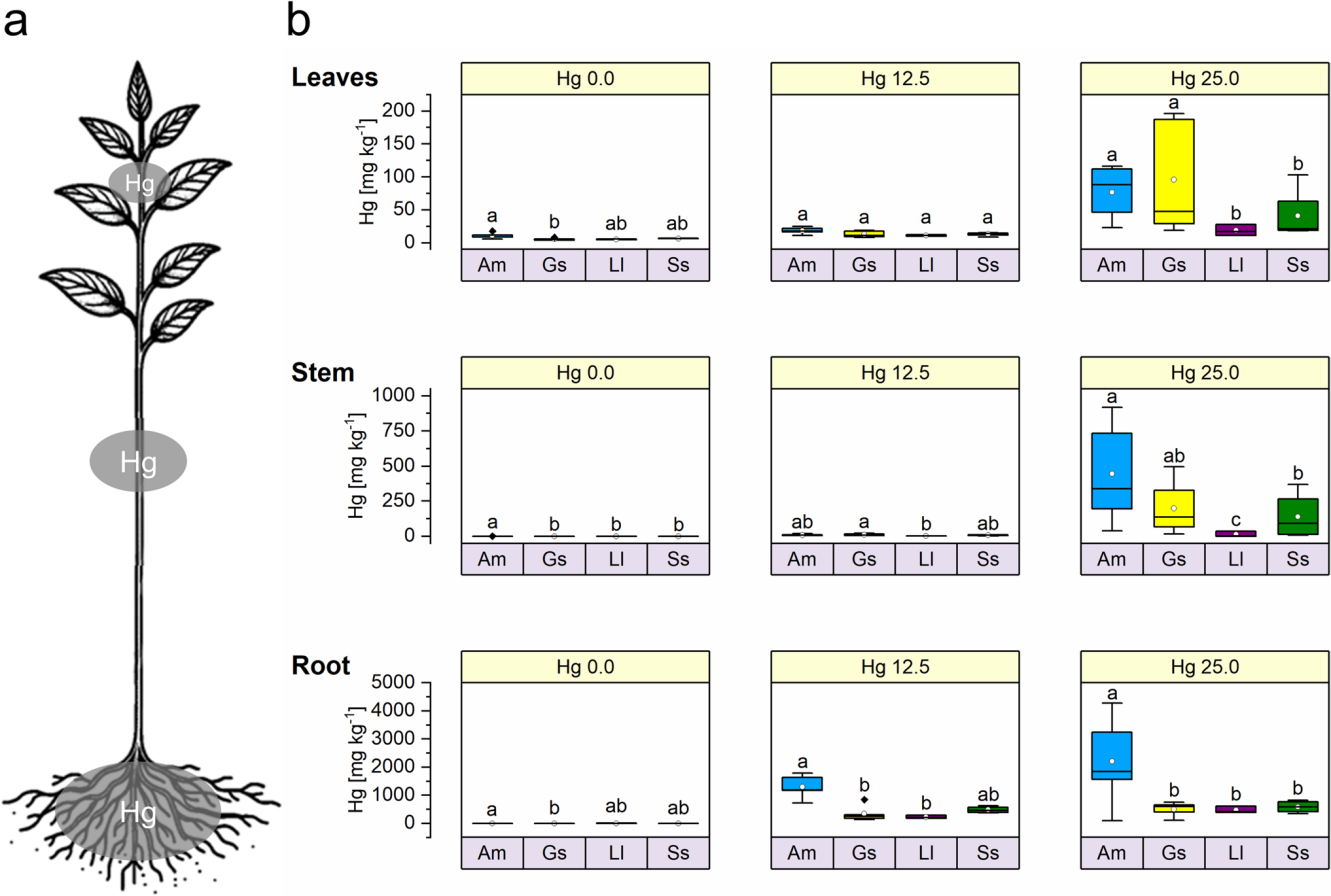
**a** Scheme of total mercury accumulation in leaves, stem, and root. **b** Data on the mercury accumulation per tissue for *A. mangium* (Am, blue), *G. sepium* (Gs, yellow), *L. leucocephala* (Ll, purple), and *S. siamea* (Ss, green). Significant differences in the effects of mercury treatment are indicated by different letters (LSD, *p* ≤ *0.05*). Statistical analysis was performed individually for each box plot diagram. The box plots represent untransformed data, while the statistical analyses were performed on log-transformed data

Our analysis of mercury accumulation across different plant components (roots, stems and leaves), shows that the highest concentrations consistently occurred in the root system, irrespective of the species studied (Fig. 1). Mercury accumulation in the trees followed the treatment with lowest concentrations at the 12.5 Hg treatment and highest at the 25 Hg treatment (Fig. 1b). The 12.5 Hg treatment did not lead to high Hg accumulation in the leaves, stems, or roots, except for the stems of *A. mangium*, which showed high Hg accumulation (Fig. 1b). The 25 Hg treatment showed accumulation in all tree species. In the leaves, highest Hg concentration was detected in *G. sepium* with 95.8 mg kg^−1^ and in *A. mangium* with 77.0 mg Hg kg^−1^. Stems of *A. mangium* (445 mg Hg kg^−1^) and *G. sepium* (360.3 mg Hg kg^−1^) accumulated highest mercury concentrations. *L. leucocephala* accumulated the lowest mercury concentration in both leaves and stem. Mercury accumulation in the above-ground biomass has a clear pattern: Am > Gs > Ss > Ll (Fig. 1b). Consideration of the increase factor of mercury accumulation shows that a doubling of mercury treatment leads to a multiple increase in mercury accumulation (Table 1). This phenomenon is particularly evident in the stems, with *A. mangium* exhibiting a 49-fold higher Hg accumulation in Hg 25.0 than in Hg 12.5.

**Table 1:** Increase factor of mercury accumulation when comparing Hg 25.0 and Hg 12.5 in leaves, stems, and roots of *A. mangium*, *G. sepium*, *L. leucocephala*, and *S. siamea*

	*A. mangium*	*G. sepium*	*L. leucocephala*	*S. siamea*
Leaves	4	7	2	3
Stem	49	33	5	20
Root	2	2	2	1

### Plant physiological responses to mercury accumulation

Table 2 displays the total dry weight, mean assimilation rate, and stem growth for each tree species. *G. sepium* showed significant and particularly pronounced reduction in assimilation rate, stem height, and total dry weight with increasing mercury treatment. *A. mangium* showed a significant decrease in assimilation rate and stem height, but total dry weight remained unchanged. *L. leucocephala* and *S. siamea* appeared resistant to mercury effects on assimilation, stem height, and dry weight, with stable assimilation rates higher mercury concentrations.

**Table 2:** Total dry weight, mean assimilation rate per plant species and stem growth over the mercury treatment period (8 w). Significant differences of treatment effects indicated by different letters (LSD, *p* ≤ *0.05*). Statistical analysis was performed individually for each tree species

Species	Mercury-treatment (mg Hg kg^−1^)	Mean assimilation (µmol m^2^ s^1^)		Stem growth (cm)	Total dry weight (g per plant)
	0.0		11.1 (± 0.7)^a^	11.9 (± 1.7)^a^	3.31 (± 0.5)^a^
*A. mangium*	12.5		8.9 (± 0.6)^ab^	11.6 (± 1.8)^a^	3.91 (± 0.3)^a^
	25.0		7.9 (± 1.9)^b^	6.6 (± 2.1)^b^	3.19 (± 0.9)^a^
	0.0		5.1 (± 0.1)^a^	9.3 (± 0.5)^a^	7.88 (± 0.2)^a^
*G. sepium*	12.5		3.6 (± 0.8)^ab^	5.4 (± 1.4)^ab^	5.62 (± 0.9)^b^
	25.0		1.5 (± 0.8)^b^	2.4 (± 1.7)^b^	4.54 (± 0.9)^b^
	0.0		5.5 (± 0.5)^a^	26.4 (± 2.5)^a^	11.35 (± 0.2)^a^
*L. leucocephala*	12.5		5.5 (± 1.0)^a^	24.4 (± 3.3)^a^	10.01 (± 0.6)^a^
	25.0		6.4 (± 0.3)^a^	21.7 (± 3.4)^a^	9.46 (± 1.2)^a^
	0.0		6.8 (± 1.0)^a^	6.6 (± 0.6)^a^	6.23 (± 0.6)^a^
*S. siamea*	12.5		7.0 (± 0.6)^a^	5.9 (± 0.6)^a^	6.80 (± 0.2)^a^
	25.0		7.6 (± 1.0)^a^	4.9 (± 0.4)^a^	6.77 (± 1.5)^a^

### Phytoremediation assessment indicators

The tolerance index (TI), calculated based on dry weight, was determined for each plant species across different treatments (Table 3). *A. mangium* exhibited a trend in dry weight accumulation at Hg 12.5 and a slight decline at Hg 25 but without statistical significance. The resulting tolerance index exceeding 1 indicated resilience to the medium mercury treatment, while a value slightly below 1 suggested a stress response at high mercury treatment. Furthermore, the ‘TI mean’ for *A. mangium* was 1.07 and with this the highest value among the four tree species indicating highest mercury tolerance. The dry mass of *S. siamea* was relatively unaffected at Hg 25 or increased at Hg 12.5 compared to control (Table 2). Therefore, the resulting TI values were in the range of 1 for both mercury treatments suggesting no harming effect of mercury on *S. siamea*. *G. sepium* exhibited the most adverse reaction to the presence of mercury, as indicated by reduced total dry weight (Table 2) and high TF of 35.4 (Table 3). The ‘TI mean’ of *L. leucocephala* was 0.86, which indicated a medium resistance range compared to the other plant species (Table 3).

**Table 3:** Translocation factor (TF), bioaccumulation factor (BAF) and tolerance index (TI) per plant species and mercury treatment. BAF mean and TI mean represent the mean value per species averaged over the levels of the mercury treatment. Significant differences of mercury treatment effects are indicated by different letters (LSD, *p* ≤ 0.05)

Species	Mercury-treatment (mg kg^−1^)	Translocation factor (TF)	Bioaccumulation factor (BAF)	BAF mean	Tolerance index (TI)	TI mean
*A. mangium*	0.0	-			-	
	12.5	0.014^a^	1.32^b^	3.45^a^	1.18^a^	1.07^a^
	25.0	0.106^b^	5.57^a^		0.96^ac^	
*G. sepium*	0.0	-			-	
	12.5	0.043^a^	0.97^b^	4.12^a^	0.71^bc^	0.65^b^
	25.0	0.354^b^	7.27^a^		0.58^c^	
*L. leucocephala*	0.0	-			-	
	12.5	0.024^a^	0.47^a^	0.54^b^	0.88^ac^	0.86^ab^
	25.0	0.028^a^	0.61^a^		0.83^ac^	
*S. siamea*	0.0	-			-	
	12.5	0.027^a^	0.97^a^	3.39^a^	1.09^ab^	1.04^a^
	25.0	0.087^b^	2.24^a^		1.09^ab^	

None of the tree species had a mercury translocation factor (TF) greater than 1.0 (Table 3). For *A. mangium* and *S. siamea*, the differences in TF between treatments were highly significant (*p* < 0.001, data not shown) with highest TF values compared to the other tree species.

The bioaccumulation factor (BAF) is a metric used to calculate the accumulation of mercury in above-ground biomass (shoot) relative to the concentration of mercury in the substrate (Table 3). Except for *L. leucocephala*, the BAF increased with increasing mercury treatment (Table 3). Notably, the highest values were observed for *A. mangium* and *G. sepium* (Table 3).

### Mycorrhizal colonization

**Fig. 2:**
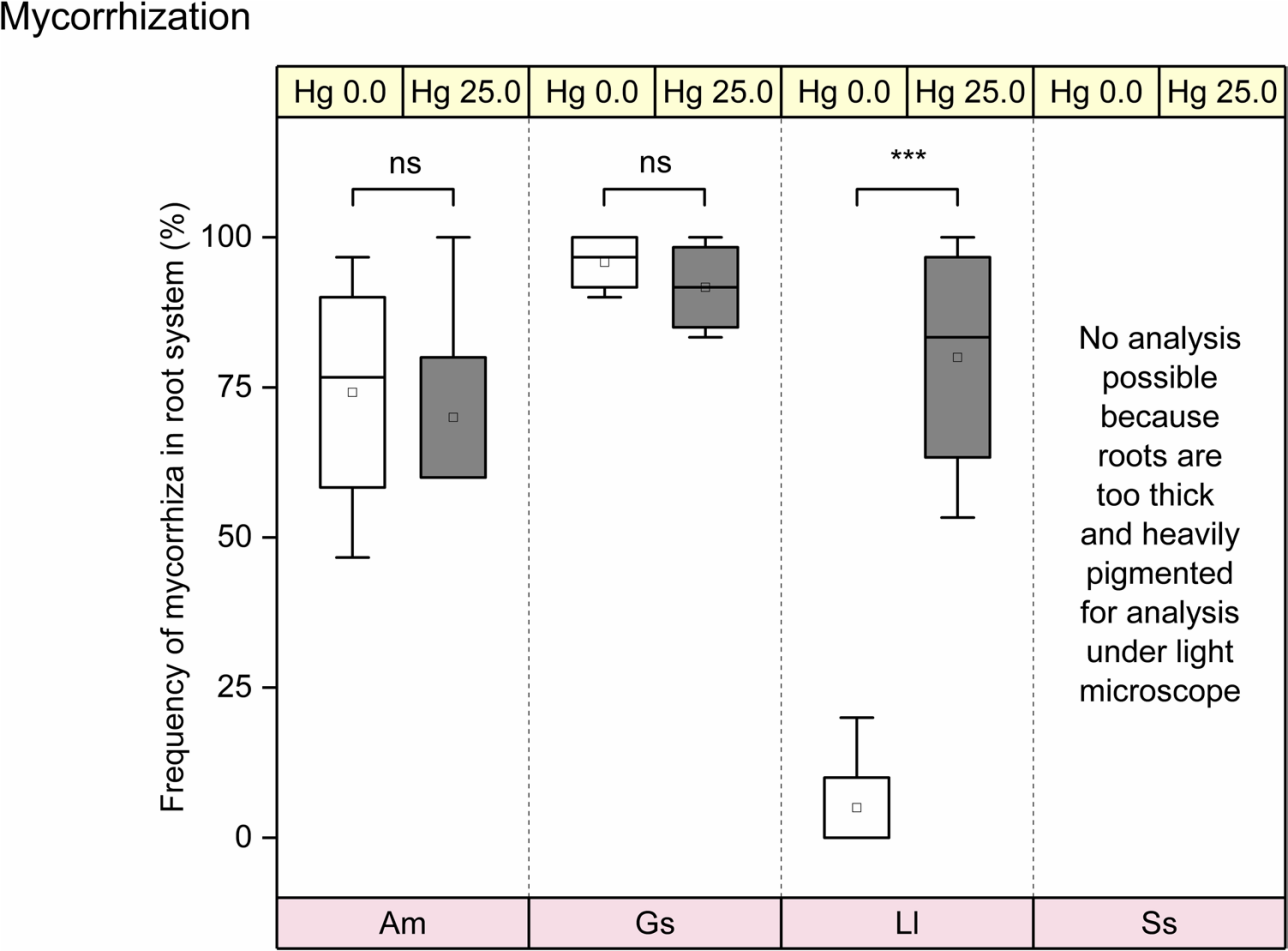
Percentage of mycorrhizal colonization in the root system of plants treated with arbuscular mycorrhizal fungi (Am = *Acacia mangium*, Gs = *Gliricidia sepium*, Ll = *Leucaena leucocephala*, Ss = *Senna siamea*) based on Trouvelot's et al. (1986) method in relation to mercury exposure (Hg 0, control, Hg 25, 25 mg Hg kg.^−1^). The roots of *S. siamea* could not be analyzed because they were too thick and heavily pigmented for analysis under the light microscope. Asterisks indicate a significant difference between Hg 0 and Hg 25 treatments (LSD,*** *p* ≤ *0.001*)

*A. mangium* and *G. sepium* had a successful inoculation with a high frequency of mycorrhizae in the root system without significant differences between control and mercury treatment (Fig. 2). However, *L. leucocephala* showed a very low mycorrhizal colonization rate in the control while the mercury-treated plants showed high inoculation capacity. The roots of *S. siamea* were too thick and heavily pigmented to be analyzed for mycorrhizal colonization under the light microscope according to the method of Trouvelot et al. (1986).

### Mercury accumulation depending on arbuscular mycorrhizal fungi

The highest mercury concentrations in aboveground biomass were found in leaves with 202 mg kg^−1^ and in stem with 203 mg kg^−1^ of AMF-treated *A. mangium* (Fig. 2). However, inoculation with AMF showed no significant effect on mercury accumulation in any of the tree species. Mercury accumulation in *G. sepium* and *L. leucocephala* was low in all plant tissues and seemed to be further reduced by inoculation with AMF, although without significant differences. The trends of mercury accumulation in the ‘mercury-concentration experiment’ were confirmed in the ‘mercury-AMF experiment’. The highest mercury concentration was always detected in *A. mangium,* the lowest in *L. leucocephala.*

### Physiological responses to mercury and arbuscular mycorrhizal fungi

The Mercury-AMF experiment aimed to assess different plant physiological parameters such as photosynthesis as indicated by the assimilation rate at week 6 (Table 4). The assimilation of *A. mangium* was significantly reduced by mercury and was increased by inoculation with AMF. The assimilation of *G. sepium* was also significantly reduced by mercury. However, *G. sepium* plants inoculated with arbuscular mycorrhizal fungi (AMF) exhibited a reduction in assimilation due to mercury treatment, though this decrease was not statistically significant. (Table 4). *L. leucocephala* had no significant change in assimilation by either mercury or AMF. In contrast, *S. siamea* had a significant reduction in assimilation in response to mercury, this was mitigated by AMF. However, neither mercury nor AMF showed a significant effect on the total dry weight of *A. mangium* (Table 4).

**Table 4:** Effect of arbuscular mycorrhizal fungi inoculation (AMF = AMF-inoculated; x = control) on mercury tolerance. Assimilation and total plant dry weight were evaluated 6 w after mercury application; bioaccumulation factor (BAF) and tolerance index (TI). Differences of significance are denoted by varying letters (LSD, *p* ≤ *0.05*). Statistical analysis was performed separately for each plant species for assimilation and total dry weight. TI and BAF compares all treatments with each other

Species	Microorganism	Mercury-treatment (mg Hg kg^−1^)	Assimilation (µmol m^2^ s^1^)	Total dry weight (g)	Tolerance index (TI)	Bioaccumulation factor (BAF)
*A. mangium*	x	0.0	15.1 (± 2.8)^a^	1.7^a^	0.85^a^	
	x	25.0	8.4 (± 2.5)^b^	1.4^a^		6.0 (± 1.0)^a^
	AMF	0.0	18.5 (± 2.8)^a^	1.4^a^	0.68^a^	
	AMF	25.0	11.6 (± 3.1)^ab^	1.0^a^		8.4 (± 1.7)^a^
*G. sepium*	x	0.0	4.7 (± 0.7)^a^	7.7^a^	0.72^a^	
	x	25.0	1.4 (± 0.7)^b^	5.6^b^		0.8 (± 0.1)^c^
	AMF	0.0	4.1 (± 0.8)^ab^	6.1^ab^	1.15^a^	
	AMF	25.0	2.8 (± 0.7)^ab^	7.0^ab^		0.6 (± 0.1)^cd^
*L. leucocephala*	x	0.0	8.2 (± 1.2)^a^	6.0^a^	0.84^a^	
	x	25.0	9.5 (± 1.3)^a^	5.0^a^		0.6 (± 0.2)^cd^
	AMF	0.0	9.5 (± 1.6)^a^	5.2^a^	1.00^a^	
	AMF	25.0	7.3 (± 1.3)^a^	5.2^a^		0.5 (± 0.1)^d^
*S. siamea*	x	0.0	6.4 (± 0.6)^a^	2.3^a^	1.02^a^	
	x	25.0	3.0 (± 0.5)^b^	2.3^a^		2.7 (± 0.8)^b^
	AMF	0.0	8.7 (± 1.7)^a^	2.8^a^	0.91^a^	
	AMF	25.0	5.4 (± 1.3)^ab^	2.6^a^		2.5 (± 1.2)^bc^

The mercury treatment showed a significant negative effect on the total dry weight (DW) of *G. sepium* (Table 4), while the inoculation with AMF did not significantly change this reduction of dry weight. The total DW of *L. leucocephala* and *S. siamea* was not significantly affected by either mercury or AMF (Table 4). Regarding the tolerance towards mercury overall, there are no significant differences in TI-values when comparing all treatments and species (Table 4). The TI-values for *A. mangium* were below 1, irrespective of the AMF-inoculation. Species and treatments with TI above 1 were *G. sepium* inoculated with AMF, and *S. siamea* without AMF. The mercury accumulated calculated as BAF showed significant differences between the tree species (Table 4), but the AMF-inoculation did not show significant effects. The highest BAF was found in *A. mangium*, the lowest in *L. leucocephala*.

## Discussion

Given the universality of the amalgamation process, similar levels of pollution, as reported by Tomiyasu et al. (2023) may be anticipated in abandoned mining sites in Ghana and other areas exposed to gold mining including amalgamation with mercury. Accordingly, our study investigated the potential of four local leguminous tree species to remediate mercury-contaminated soils. Generally, mercury accumulation in plant tissues has a complex pattern, with roots consistently showing the highest concentrations (Fig. 1), as shown in other studies (Liu et al. 2017; Moreno et al. 2005). However, the accumulation of mercury in aboveground biomass is particularly important for phytoremediation (Ali et al. 2013). Our data revealed that specifically under high mercury treatment, *A. mangium* accumulated highest concentrations in above-ground biomass, closely followed by *G. sepium* (Fig. 1). Therefore, these two species are considered as mercury accumulators. *L. leucocephala* shows comparatively low values in the aboveground biomass (Fig. 1) and may be classified as an excluder (Baker 1981).

The increase in mercury accumulation in the aboveground biomass, especially at the high mercury concentration in soil is of particular interest. Although the mercury concentration in the substrate increased twofold (from Hg 12.5 to Hg 25), the concentration in the biomass of all four tree species increased up to 49-fold in stems of *A. mangium* and sevenfold in the leaves of *G. sepium* (Table 1). In the roots, however, twofold soil mercury increase led to a twofold increase in mercury accumulation—irrespective of the tree species (Table 1). This suggests a threshold above which mercury accumulation becomes more effective but also more harmful for above-ground plant tissue. According to Baker (1981) *A. mangium* and *G. sepium* showed the characteristics of metal accumulators.

### Physiological responses and suitability for phytoremediation

The ideal candidate suitable for phytoextraction should not only accumulate high concentrations of mercury in the aboveground biomass, but also remain unharmed (Alkorta et al. 2004). The high mercury-accumulating species *A. mangium* showed a reduction in assimilation and stem height but no reduction in total dry weight (Table 2). It could be suggested that mercury did not harm the plant but led to a more stunted habitus. On the contrary, *G. sepium* was severely affected; not only did assimilation and stem height decrease with increasing mercury treatment (Table 2), moreover, four out of 10 mercury-treated plants did not survive during the experiment (data not shown). Therefore, *G. sepium* is regarded as a susceptible tree species that is unsuitable for the phytoremediation of mercury-contaminated soils. The physiology of *L. leucocephala* was not affected by mercury; however, it did not absorb a significant amount of mercury (Table 2, Fig. 1). Thus, even though the plant can likely tolerate mercury-contaminated soil, it would not qualify as an effective accumulator.

**Fig. 3:**
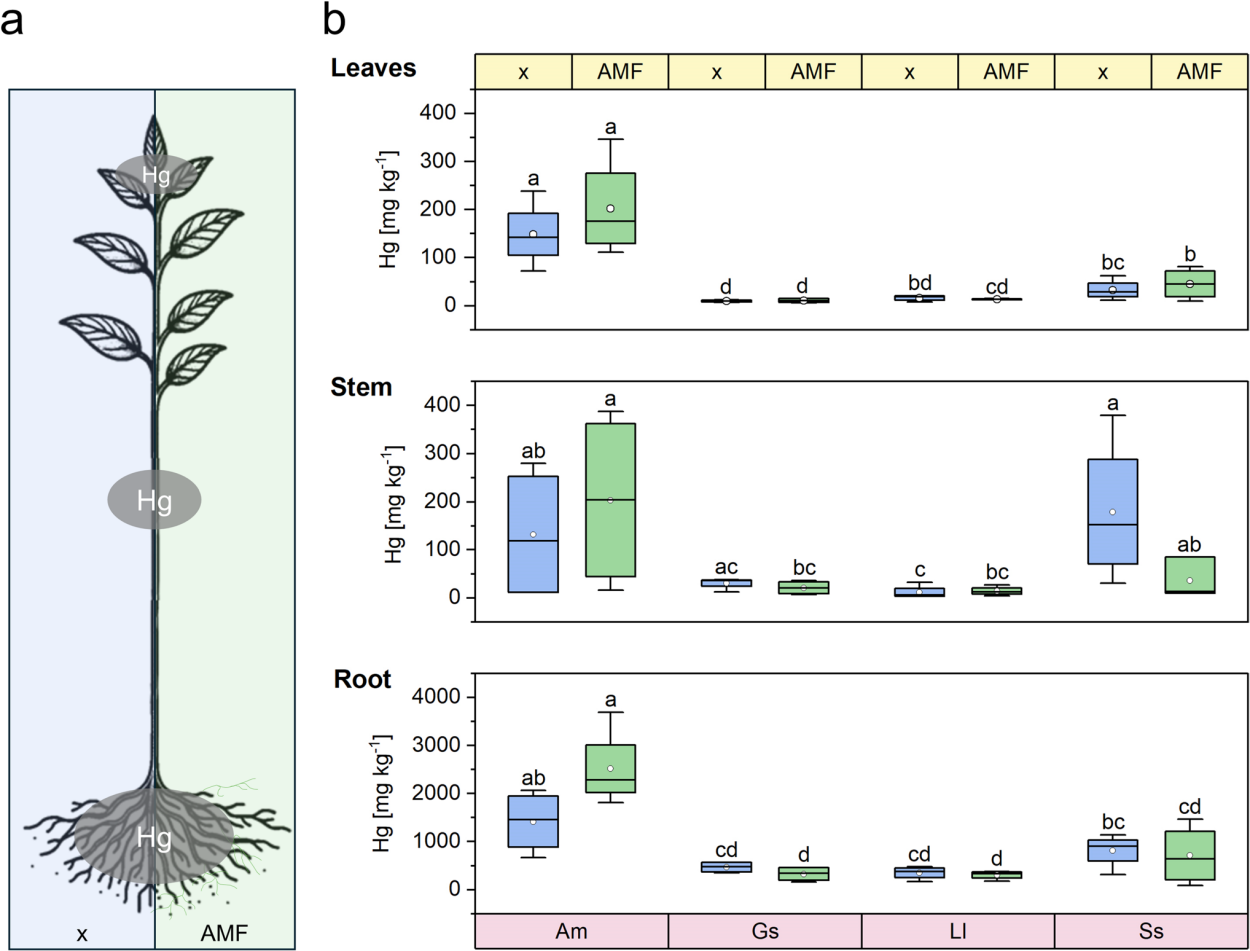
**a** Scheme of mercury accumulation in leaves, stem, and root depending on the presence or absence of arbuscular mycorrhizal fungi (AMF). **b** Mercury accumulation concentrations in the leaves, stems, and roots of plants (Am, *Acacia mangium*; Gs, *Gliricidia sepium*; Ll, *Leucaena leucocephala*; Ss, *Senna siamea*) after inoculation with arbuscular mycorrhizal fungi (AMF, AMF-inoculated; x, control). The data presented pertain solely to plants subjected to mercury treatment (25 mg Hg kg^−1^). Variations of significance are denoted by distinct letters (LSD, *p* ≤ *0.05*). Box plots, non-transformed values; statistical assessments with log-transformation

Metal remediation plant indicators have been developed to assist the comparability of different plant species for their phytoremediation potential. It is the main ambition to locate so-called hyperaccumulators, plants that accumulate heavy metals in their high yielding above-ground tissues to levels far above those found in the soil (Memon and Schröder 2009). Characteristics of hyperaccumulators are high concentration thresholds for heavy metals in plant shoots (e.g., 10,000 mg kg^−1^ for zinc (Zn), 1000 mg kg^−1^ for nickel (Ni), and 100 mg kg^−1^ for cadmium (Cd)) (Baker 1981; Baker and Brooks 1989). No threshold levels have yet been set for mercury. Further, the bioaccumulation calculated as bioaccumulation factor (BAF) as well as the translocation factor (TF) should be greater than 1 (Liu et al. 2020; Xun et al. 2017). Finally, the plants should be extremely tolerant to heavy metals, *i.e*. they should not show significant reduction in biomass as determined by the tolerance with a TI-value of > 1 (Diwan et al. 2010). Despite numerous studies conducted from various countries on over 200 plant species in the last two decades in search of a hyperaccumulator of mercury, none has been successfully identified so far (Liu et al. 2020). Only *Erato polymnioides* and a few other species were labeled as potential mercury hyperaccumulators (Chamba et al. 2017). However, these species are native to the rainforests of Bolivia, Colombia, Costa Rica, Ecuador, Panamá, and Peru—not to West Africa. Introducing non-native plant species can destabilize local ecosystems, as these species often lack natural predators or competitors, allowing them to spread rapidly and become invasive (Zizka et al. 2015). According to the definition, none of the four plant species meets all criteria. *G. sepium* accumulated high levels of mercury, resulting in comparatively high TF values, but suffered considerable damage and plant death due to its sensitivity to mercury, as evident from its low TI values and the reduction in assimilation (Table 2 and 3). *L. leucocephala* had the lowest mercury accumulation in the aboveground biomass (Fig. 1) and showed the lowest mercury translocation factors of all species (Table 3). However, *L. leucocephala* did not respond to the mercury treatment in any way. Neither the stem growth, dry weight, nor the assimilation was affected (Table 2, 3, and 4), suggesting that it can be classified as an excluder plant. *S. siamea* fulfilled the majority of the set requirements and demonstrated a TI above 1 and a BAF above 2 in both experiments. However, in comparison to *A. mangium*, *S. siamea* exhibited a reduced accumulation of mercury in both experiments, as well as a lower BAF (Table 3 and 4, Figs. 1 and 3). With regard to the accumulation of mercury, *A. mangium* is the species with the greatest potential for mercury phytoremediation, as it exhibits the highest concentration of mercury in plant tissues and the highest BAF in both experiments (Table 3 and 4, Figs. 1 and 3). It proved to be resistant to mercury exposure, with a TI greater than 1 in the mercury-concentration-Experiment (Diwan et al. 2010). Further, its BAF in the 25 Hg treatment was higher than in the 12.5 Hg treatment, corresponding to its increased mercury accumulation (Fig. 1, Table 3). Most importantly, *A. mangium* accumulated by far the highest concentration of mercury in above-ground tissue, even though not being a hyperaccumulator.

This differentiation among the species is of practical importance with respect to their local uses*. A. mangium* and *S. siamea* accumulated the highest concentrations of mercury but are mainly valued for timber, fuelwood, shade, and land rehabilitation rather than as fodder (Dupuy and Kanga 1990; Hegde et al. 2013). In contrast, *L. leucocephala* and *G. sepium*, both important fodder trees in West African agroforestry systems (Zayed et al. 2018; Alamu et al. 2023), accumulated comparatively little mercury in our experiments. This considerably reduces the risk of mercury transfer into the food chain and highlights the potential of combining strong accumulator species such as *A. mangium*, with robust excluder or multipurpose species such as *L. leucocephala*, in phytoremediation-oriented restoration strategies. *A. mangium* stands out as a particularly promising candidate for the phytoremediation of mercury-contaminated soils in Ghana, consistent with previous studies demonstrating its capacity to remediate heavy metal-polluted sites (Sampanpanish 2018; Rosli et al. 2021; Couic et al. 2021). The concept of diverse forests for phytoremediation is well acknowledged and a fast-growing topic in land restoration research, as reviewed by Gómez et al. (2019). The proposal here is not to exploit the forests directly for short-term gains, but to carefully manage and monitor contamination levels over several decades so that the acacia forest and other organisms can establish a stable ecosystem that facilitates the natural decontamination of anthropogenic pollution. Therefore, *A. mangium*, a promising candidate for the phytoextraction of mercury-contaminated soils, could be accompanied by *S. siamea*, which has also achieved promising results, and *L. leucocephala*, a robust and fast-growing mercury excluder that develops well on mercury-contaminated soils. *L. leucocephala* is a commonly utilized tree in the maintenance of agroforestry systems (Shelton 1998). As such, *L. leucocephala* could potentially contribute to the biodiversity of a remediating forest. Furthermore, previous studies have shown that *S. siamea*, in combination with other plants, provides significant benefits for soil remediation by accumulating heavy metals, improving soil structure and promoting fertility, thus supporting sustainable plant growth in heavy metal-contaminated soils (Vanlauwe et al. 2005; Kusumaningtyas et al. 2021).

### Physiological responses to mercury and arbuscular mycorrhizal fungi

Research indicated that heavy metals typically decrease the mycorrhizal colonization; however, in our case (Fig. 2) the opposite effect was observed, a finding in line with earlier work (Schneider et al. 2017; Garcia et al. 2020). In our study, it is not possible to predict a general trend as to whether mercury accumulation is generally increased or decreased by inoculation with AMF, as no significant difference was found (Fig. 3). Previous findings indicate that inoculation with AMF can lead to both a decrease or an increase in the uptake of mercury by plants (Debeljak et al. 2018; Li et al. 2023). Although inoculation with AMF was successful, it did not result in a significant difference in assimilation or total dry weight as in other studies (Frosi et al. 2016; Estrada-Luna and Davies 2003). Nevertheless, it seems that AMF exerts a ‘buffer effect’ on the sensitivity of plants to mercury, as the reduction in assimilation in less pronounced in the mercury-accumulating species *A. mangium*, *G. sepium* and *S. siamea* (Table 4). This is consistent with previous findings that AMF can increase a plant's tolerance of heavy metals by binding toxic elements and promoting nutrient and antioxidant responses (Hildebrandt et al. 2007; Dhalaria et al. 2020). Comparing the two experiments, *A. mangium* was able to achieve an even higher mercury accumulation in the mercury-AMF experiment than in the mercury-concentration experiment (Tables 3 and 4). Thus, the inoculation with AMF seemed to increase the BAF of *A. mangium*, but the TI of *A. mangium* in the mercury-AMF experiment was below 1 both with and without AMF (Table 4). In contrast, the BAF of *G. sepium* in the mercury-AMF experiment was much lower than in the mercury-concentration experiment (Table 2 and 4). This was probably due to the reduced mercury accumulation, whereby the TI of *G. sepium* in mercury-AMF was higher than 1. In *A. mangium* and *G. sepium*, the age of the plant and the duration of mercury exposure appeared to influence the process of mercury accumulation. The influence of the duration of mercury exposure on accumulation has already been observed in other research projects (Xun et al. 2017; Millhollen et al. 2006). The mechanisms underlying the ageing effect of plants on mercury accumulation are a subject for future research.

## Conclusion

Although it cannot be classified as a hyperaccumulator this study identified *A. mangium* as the most promising species for phytoremediation of mercury-contaminated soils in former gold mining areas. It accumulates the highest concentrations of mercury and shows only minor signs of mercury toxicity making it well suitable for planting on areas with former gold mining. Compared to *A. mangium S. siamea* exhibited lower (moderate) mercury accumulation without physiological impairment. *L. leucocephala* did not accumulate mercury, but also demonstrated no adverse physiological effects and could play an important role as a mercury excluder, thereby promoting biodiversity and ecosystem stability. *G. sepium* showed moderate mercury uptake but suffered considerable physiological damage. Although inoculation with AMF did not significantly increase mercury uptake, it appeared to mitigate physiological stress, supporting plant health rather than directly enhancing mercury removal. These findings support the strategic use of diverse plant species in ecological restoration to optimize mercury removal and promote a stable ecosystem. Further research is needed to identify other suitable plant species that meet all the criteria for mercury hyperaccumulation and can be effectively used in phytoremediation strategies.

## Supplementary Information

Below is the link to the electronic supplementary material.

ESM 1 (JPG 429 KB)

ESM 2 (JPG 598 KB)

ESM 3 (JPG 5.11 MB)

ESM 4 (JPG 1.24 MB)

ESM 5 (JPG 1.34 MB)

ESM 6 (JPG 1.31 MB)

ESM 7 (JPG 1.15 MB)

## Data Availability

The datasets generated during and/or analyzed during the current study are available from the corresponding author on reasonable request.

## References

[CR1] Alamu EO, Adesokan M, Fawole S, Maziya-Dixon B, Mehreteab T, Chikoye D (2023) *Gliricidia sepium* (Jacq.) walp applications for enhancing soil fertility and crop nutritional qualities: a review. Forests 14:1–14. 10.3390/f1403063540013316

[CR2] Ali H, Khan E, Sajad MA (2013) Phytoremediation of heavy metals-concepts and applications. Chemosphere 91(7):869–881. 10.1016/j.chemosphere.2013.01.075 23466085 10.1016/j.chemosphere.2013.01.075

[CR3] Alkorta I, Hernandez-Allica J, Becerril JM, Amezaga I, Albizu I, Garbisu C (2004) Recent findings on the phytoremediation of soils contaminated with environmentally toxic heavy metals and metalloids such as zinc, cadmium, lead, and arsenic. Rev Environ Sci Biotechnol 3(1):71–90. 10.1023/B:RESB.0000040059.70899.3d

[CR4] Aryee BNA, Ntibery BK, Atorkui E (2003) Trends in the small-scale mining of precious minerals in Ghana: a perspective on its environmental impact. J Clean Prod 11(2):131–140. 10.1016/S0959-6526(02)00043-4

[CR5] Baker AJM (1981) Accumulators and excluders - strategies in the response of plants to heavy metals. J Plant Nutr 3(1–4):643–654. 10.1080/01904168109362867

[CR6] Baker AJM, Brooks RR (1989) Terrestrial higher plants which hyperaccumulate metallic elements - a review of their distribution, ecology and phytochemistry. Biorecovery 1(2):81–126

[CR7] Boateng Y (2014) Phytoremediation of heavy metal contaminated soil using *Leucaena leucocephala*. A case study at anglogold Ashanti Obuasi Ghana. Dissertation, Kwame Nkrumah University

[CR8] Boateng DO, Codjoe FNY, Ofori J (2014) Impact of illegal small scale mining (galamsey) on cocoa production in Atiwa district of Ghana. Int J Adv Agric Res 2:89–99. 10.1080/10962247.2014.905509

[CR9] Chamba I, Rosado D, Kalinhoff C, Thangaswamy S, Sánchez-Rodríguez A, Gazquez MJ (2017) *Erato polymnioides* – a novel Hg hyperaccumulator plant in Ecuadorian rainforest acid soils with potential of microbe-associated phytoremediation. Chemosphere 188:633–641. 10.1016/j.chemosphere.2017.08.160 28918247 10.1016/j.chemosphere.2017.08.160

[CR10] Couic E, Tribondeau A, Alphonse V, Livet A, Grimaldi M, Bousserrhine N (2021) The impact of ecological restoration on biogeochemical cycling and mercury mobilization in anoxic conditions on former mining sites in french guiana. Microorganisms 9:1–19. 10.3390/microorganisms90817020 10.3390/microorganisms9081702 PMC8400979 34442781

[CR11] Debeljak M, van Elteren JT, Špruk A, Izmer A, Vanhaecke F, Vogel-Mikuš K (2018) The role of arbuscular mycorrhiza in mercury and mineral nutrient uptake in maize. Chemosphere 212:1076–1084. 10.1016/j.chemosphere.2018.08.147 30286537 10.1016/j.chemosphere.2018.08.147

[CR12] Dhalaria R, Kumar D, Kumar H, Nepovimova E, Kuca K, Islam MT, Verma R (2020) Arbuscular mycorrhizal fungi as potential agents in ameliorating heavy metal stress in plants. Agronomy 10. 10.3390/agronomy10060815

[CR13] Diwan H, Ahmad A, Iqbal M (2010) Uptake-related parameters as indices of phytoremediation potential. Biologia (Bratisl) 65(6):1004–1011. 10.2478/s11756-010-0106-7

[CR14] Donkor AK, Nartey VK, Bonzongo JC, Adotey DK (2009) Artisanal mining of gold with mercury in Ghana. West African J Appl Ecol 9(1). 10.4314/wajae.v9i1.45666

[CR15] Dupuy B, Kanga N (1990) Sylviculture de l’Acacia mangium en basse Côte d’Ivoire. Bois Forêts des Trop 225:24–32. 10.19182/BFT1990.225.A19659

[CR16] Estrada-Luna AA, Davies FT (2003) Arbuscular mycorrhizal fungi influence water relations, gas exchange, abscisic acid and growth of micropropagated chile ancho pepper (*Capsicum annuum*) plantlets during acclimatization and post-acclimatization. J Plant Physiol 160(9):1073–1083. 10.1078/0176-1617-00989 14593809 10.1078/0176-1617-00989

[CR17] FAO (2024) Food and Agriculture Organization of the United Nations. Employment indicators: agriculture. Share of employment in agriculture in total employment. https://www.fao.org/faostat/en/#data/OEA. Accessed 29 Oct 2024

[CR18] Ferrol N, Tamayo E, Vargas P (2016) The heavy metal paradox in arbuscular mycorrhizas: from mechanisms to biotechnological applications. J Exp Bot 67(22):6253–6565. 10.1093/jxb/erw403 27799283 10.1093/jxb/erw403

[CR19] Festin ES, Tigabu M, Chileshe MN, Syampungani S, Odén PC (2019) Progresses in restoration of post-mining landscape in Africa. J for Res 30:381–396. 10.1007/s11676-018-0621-x

[CR20] Frérot H, Lefèbvre C, Gruber W, Collin C, Dos Santos A, Escarré J (2006) Specific interactions between local metallicolous plants improve the phytostabilization of mine soils. Plant Soil 282(1–2):53–65. 10.1007/s11104-005-5315-4

[CR21] Frosi G, Barros VA, Oliveira MT, Cavalcante UMT, Maia LC, Santos MG (2016) Increase in biomass of two woody species from a seasonal dry tropical forest in association with amf with different phosphorus levels. Appl Soil Ecol 102:46–52. 10.1016/j.apsoil.2016.02.009

[CR22] Garbisu C, Alkorta I (2001) Phytoextraction: a cost-effective plant-based technology for the removal of metals from the environment. Bioresource Technol 77(3):229–236. 10.1016/S0960-8524(00)00108-5 10.1016/s0960-8524(00)00108-5 11272009

[CR23] Garcia KGV, Mendes Filho PF, Pinheiro JI, do Carmo JF, de Araújo AP, Pereira, Martins CM, Abreu MGP, Filho J (2020) Attenuation of manganese-induced toxicity in *Leucaena leucocephala* colonized by arbuscular mycorrhizae. Water Air Soil Poll 231(22). 10.1007/s11270-019-4381-9

[CR24] Garg N, Singh S (2018) Arbuscular mycorrhiza Rhizophagus irregularis and silicon modulate growth, proline biosynthesis and yield in Cajanus cajan L. Millsp. (pigeonpea) genotypes under cadmium and zinc stress. J Plant Growth Regul 37(1):46–63. 10.1007/s00344-017-9708-4

[CR25] Gómez L, Contreras A, Bolonio D, Quintana J, Oñate-Sánchez L, Merino I (2019) Phytoremediation with trees. Adv Bot Res (89). 10.1016/bs.abr.2018.11.010. Elsevier Ltd

[CR26] Hegde M, Palanisamy K, Yi JS (2013) *Acacia mangium* Willd. - a fast growing tree for tropical plantation. J for Environ Sci 29:1–14. 10.7747/jfs.2013.29.1.1

[CR27] Hildebrandt U, Regvar M, Bothe H (2007) Arbuscular mycorrhiza and heavy metal tolerance. Phytochemistry 68:139–146. 10.1016/j.phytochem.2006.09.023 17078985 10.1016/j.phytochem.2006.09.023

[CR28] Hughes JB, Shanks J, Vanderford M, Lauritzen J, Bhadra R (1997) Transformation of TNT by aquatic plants and plant tissue cultures. Environ Sci Technol 31(1):266–271. 10.1021/es960409h

[CR29] Kusumaningtyas VA, Azizah HN, Murniati A, Budiman S, Sujono H, Hardian A, Melina M, Setiawati T, Setiawati D (2021) The potency of Cassia siamea as phytostabilization in post-mining land reclamation. IOP Conf Ser Earth Environ Sci 882. 10.1088/1755-1315/882/1/012072

[CR30] Mei L, Zhu Y, Zhang X, Zhou X, Zhong Z, Li H, Li X, Li X, Daud MK, Chen J, Zhu S (2021) Mercury-induced phytotoxicity and responses in upland cotton (*Gossypium hirsutum* L.) Seedlings. 2(May):1–15. 10.20944/preprints202105.0228.v1 10.3390/plants10081494 PMC8398479 34451539

[CR31] Lenti K, Fodor F, Böddi B (2002) Mercury inhibits the activity of the NADPH: protochlorophyllide oxidoreductase (por). Photosynthetica 40(1):145–151. 10.1023/A:1020143602973

[CR32] Li X, Zhou M, Shi F, Meng B, Liu J, Mi Y, Dong C, Su H, Liu X Wang F, Wei Y (2023) Influence of arbuscular mycorrhizal fungi on mercury accumulation in rice (*Oryza sativa* L.): from enriched isotope tracing perspective. Ecotoxicol Environ Safety 255(11–2022):114776. 10.1016/j.ecoenv.2023.114776 10.1016/j.ecoenv.2023.114776 36931088

[CR33] Liu Z, Chen B, Wang L, Urbanovich O, Nagorskaya L, Li X, Tang L (2020) A review on phytoremediation of mercury contaminated soils. J Hazard Mater 400(6):123138. 10.1016/j.jhazmat.2020.123138 32947735 10.1016/j.jhazmat.2020.123138

[CR34] Liu Z, Wang L-A, Xu J, Ding S, Feng X, Xiao H (2017) Effects of different concentrations of mercury on accumulation of mercury by five plant species. Ecol Eng 106:273–278. 10.1016/j.ecoleng.2017.05.051

[CR35] Macedo MO, Resende AS, Garcia PC, Boddey RM, Jantalia CP, Urquiaga S, Campello EFC, Franco AA (2008) Changes in soil C and N stocks and nutrient dynamics 13 years after recovery of degraded land using leguminous nitrogen-fixing trees. Forest Ecol Manag 255(5–6):1516–1524. 10.1016/j.foreco.2007.11.007

[CR36] Memon AR, Schröder P (2009) Implications of metal accumulation mechanisms to phytoremediation. Environ Sci Pollut Res Int 16(2):162–175. 10.1007/s11356-008-0079-z 19067014 10.1007/s11356-008-0079-z

[CR37] Mertens J, Vervaeke P, Schrijver AD, Luyssaert S (2004) Metal uptake by young trees from dredged brackish sediment: limitations and possibilities for phytoextraction and phytostabilisation. Sci Total Environ 326(1–3):209–215. 10.1016/j.scitotenv.2003.12.010 15142776 10.1016/j.scitotenv.2003.12.010

[CR38] Millhollen AG, Gustin MS, Obrist D (2006) Foliar mercury accumulation and exchange for three tree species. Environ Sci Technol 40(19):6001–6006. 10.1021/es0609194 17051791 10.1021/es0609194

[CR39] Moreno FN, Anderson CWN, Stewart RB, Robinson BH, Ghomshei M, Meech JA (2005) Induced plant uptake and transport of mercury in the presence of sulphur-containing ligands and humic acid. New Phytol 166(2):445–454. 10.1111/j.1469-8137.2005.01361.x 15819909 10.1111/j.1469-8137.2005.01361.x

[CR40] Motaharpoor Z, Taheri M, Nadian H (2019) *Rhizophagus irregularis* modulates cadmium uptake, metal transporter, and chelator gene expression in *Medicago sativa*. Mycorrhiza 29(4):389–395. 10.1007/s00572-019-00900-7 31218402 10.1007/s00572-019-00900-7

[CR41] OEC (2024) Country profile Ghana. https://oec.world/en/profile/country/gha#latest-data. Accessed 3 May 2024

[CR42] Osei-Tutu G, Abunyewa AA, Dawoe EK, Agbenyega O, Barnes RV (2018) Effect of multipurpose trees and shrubs on degraded mined-out soil in a semi-deciduous forest zone of West Africa. L Degrad Dev 29:3432–3439. 10.1002/ldr.3110

[CR43] Padmavathiamma PK, Li LY (2007) Phytoremediation technology: hyper-accumulation metals in plants. Water Air Soil Pollut 184(1–4):105–126. 10.1007/s11270-007-9401-5

[CR44] Pirsarandib Y, Hassanpouraghdam MB, Rasouli F, Aazami MA, Puglisi I, Baglieri A (2022) Phytoremediation of soil contaminated with heavy metals via arbuscular mycorrhiza (*Funneliformis mosseae*) inoculation ameliorates the growth responses and essential oil content in lavender (*Lavandula angustifolia* L.). Agronomy 12:1221. 10.3390/agronomy12051221

[CR45] Rosli RA, Harumain ZAS, Zulkalam MF, Hamid AAA, Sharif MF, Mohamad MAN, Noh AL, Shahari R (2021) Phytoremediation of arsenic in mine wastes by *Acacia mangium*. Remediation 31:49–59. 10.1002/rem.21688

[CR46] Salazar MJ, Menoyo E, Faggioli V, Geml J, Cabello M, Rodriguez JH, Marro N, Pardo A, Pignata ML, Becerra AG (2018) Pb accumulation in spores of arbuscular mycorrhizal fungi. Sci Total Environ 643:238–246. 10.1016/j.scitotenv.2018.06.199 29936165 10.1016/j.scitotenv.2018.06.199

[CR47] Sampanpanish P (2018) Arsenic, manganese, and cyanide removal in a tailing storage facility for a gold mine using phytoremediation. Remediation 28:83–89. 10.1002/rem.21563

[CR48] Schneider J, Bundschuh J, de Melo Rangel W, Guilherme LRG (2017) Potential of different AM fungi (native from As-contaminated and uncontaminated soils) for supporting *Leucaena leucocephala* growth in As-contaminated soil. Environ Pollut 224:125–135. 10.1016/j.envpol.2017.01.071 28214191 10.1016/j.envpol.2017.01.071

[CR49] Schueler V, Kuemmerle T, Schröder H (2011) Impacts of surface gold mining on land use systems in western Ghana. Ambio 40(5):528–539. 10.1007/s13280-011-0141-9 21848141 10.1007/s13280-011-0141-9 PMC3357810

[CR50] Shabani L, Sabzalian MR, Mostafavi pour S (2016) Arbuscular mycorrhiza affects nickel translocation and expression of ABC transporter and metallothionein genes in *Festuca arundinacea*. Mycorrhiza 26(1):67–76. 10.1007/s00572-015-0647-2 26041568 10.1007/s00572-015-0647-2

[CR51] Shelton HM (1998) The *Leucaena* genus: New opportunities for agriculture. Proc Leucaena-Adapt Qual Farming Sys, Hanoi, Vietnam, 9–14 February 1998: 15–25. ISBN: 1–86320–232–3

[CR52] Sheoran V, Sheoran AS, Poonia P (2011) Role of hyperaccumulators in phytoextraction of metals from contaminated mining sites: a review. Crit Rev Environ Sci Technol 41(2):168–214. 10.1080/10643380902718418

[CR53] Singh G, Pankaj U, Chand S, Verma RK (2019) Arbuscular mycorrhizal fungi-assisted phytoextraction of toxic metals by Zea mays L. from tannery sludge. Soil Sediment Contam 28(8):729–746. 10.1080/15320383.2019.1657381

[CR54] Tetteh EN, Ampofo KT, Logah V (2015) Adopted practices for mined land reclamation in Ghana: a case study of Anglogold Ashanti Iduapriem mine Ltd. J Sci Technol 35(2):77–88. 10.4314/just.v35i2.8

[CR55] Tomiyasu T, Yasumatsu S, Kodamatani H, Kanzaki R, Takenaka C, Murao S, Miyagawa S, Nonaka K, Ikeguchi A, Navarrete IA (2023) The dynamics of mercury around an artisanal and small-scale gold mining area, Camarines Norte, Philippines. Environ Sci Pollut Res Int 30(8):20052–20064. 10.1007/s11356-022-23497-5 36251184 10.1007/s11356-022-23497-5

[CR56] Trouvelot A, Kough JL, Gianinazzi-Pearson V (1986) Mesure du taux de mycorhization VA d’un systeme radiculaire. Recherche de methods d’estimation ayant une signification fonctionnelle. In: Gianinazzi-Pearson V, Gianinazzi S (eds) Physiological and Genetical Aspects of Mycorrhizae. INRA Press, Paris, pp 217–221

[CR57] UN Environment (2019) Global mercury assessment 2018. UN Environment Programme, Chemicals and Health Branch Geneva, Switzerland. https://wedocs.unep.org/handle/20.500.11822/27579

[CR58] Vanlauwe B, Aihou K, Tossah BK, Diels J, Sanginga N, Merckx R (2005) *Senna siamea* trees recycle Ca from a Ca-rich subsoil and increase the topsoil pH in agroforestry systems in the West African derived savanna zone. Plant Soil 269:285–296. 10.1007/s11104-004-0599-3

[CR59] Weissenhorn I, Leyval C, Belgy G, Berthelin J (1995) Arbuscular mycorrhizal contribution to heavy metal uptake by maize (*Zea mays* L.) in pot culture with contaminated soil. Mycorrhiza 5(4):245–251. 10.1007/s005720050068

[CR60] Xun Y, Feng L, Li Y, Dong H (2017) Mercury accumulation plant *Cyrtomium macrophyllum* and its potential for phytoremediation of mercury polluted sites. Chemosphere 189:161–170. 10.1016/j.chemosphere.2017.09.055 28934656 10.1016/j.chemosphere.2017.09.055

[CR61] Yon YR, Pérez LA, Carmona AM, Pérez YM, García LRM, Suárez KF, Echevarría AM (2015) Alternative stainning technique to deter- mine mycorrhizal colonization. Cultivos Tropicales 36:18–21. 10.13140/RG.2.2.10232.65287

[CR62] Yoon J, Cao X, Zhou Q, Ma LQ (2006) Accumulation of Pb, Cu, and Zn in native plants growing on a contaminated Florida site. Sci Total Environ 368(2–3):456–464. 10.1016/j.scitotenv.2006.01.016 16600337 10.1016/j.scitotenv.2006.01.016

[CR63] Zayed MZ, Sallam SMA, Shetta ND (2018) Review article on *Leucaena leucocephala* as one of the miracle timber trees. Int J Pharm Pharm Sci 10:1. 10.22159/ijpps.2018v10i1.18250

[CR64] Zizka A, Thiombiano A, Dressler S, Nacoulma BMI, Ouédraogo A (2015) The vascular plant diversity of Burkina Faso (West Africa), a quantitative and implicationanalysis s for conservation. Candollea 70:9–20. 10.15553/c2015v701a

