# Supplementary figures and images for: The potential of four legume trees for mercury phytoremediation and the role of arbuscular mycorrhizal fungi
Source: Environ Sci Pollut Res Int. 2026 Jul 11;33(23):11665–78. doi: 10.1007/s11356-026-38009-y (PMC13424149; doi:10.1007/s11356-026-38009-y)

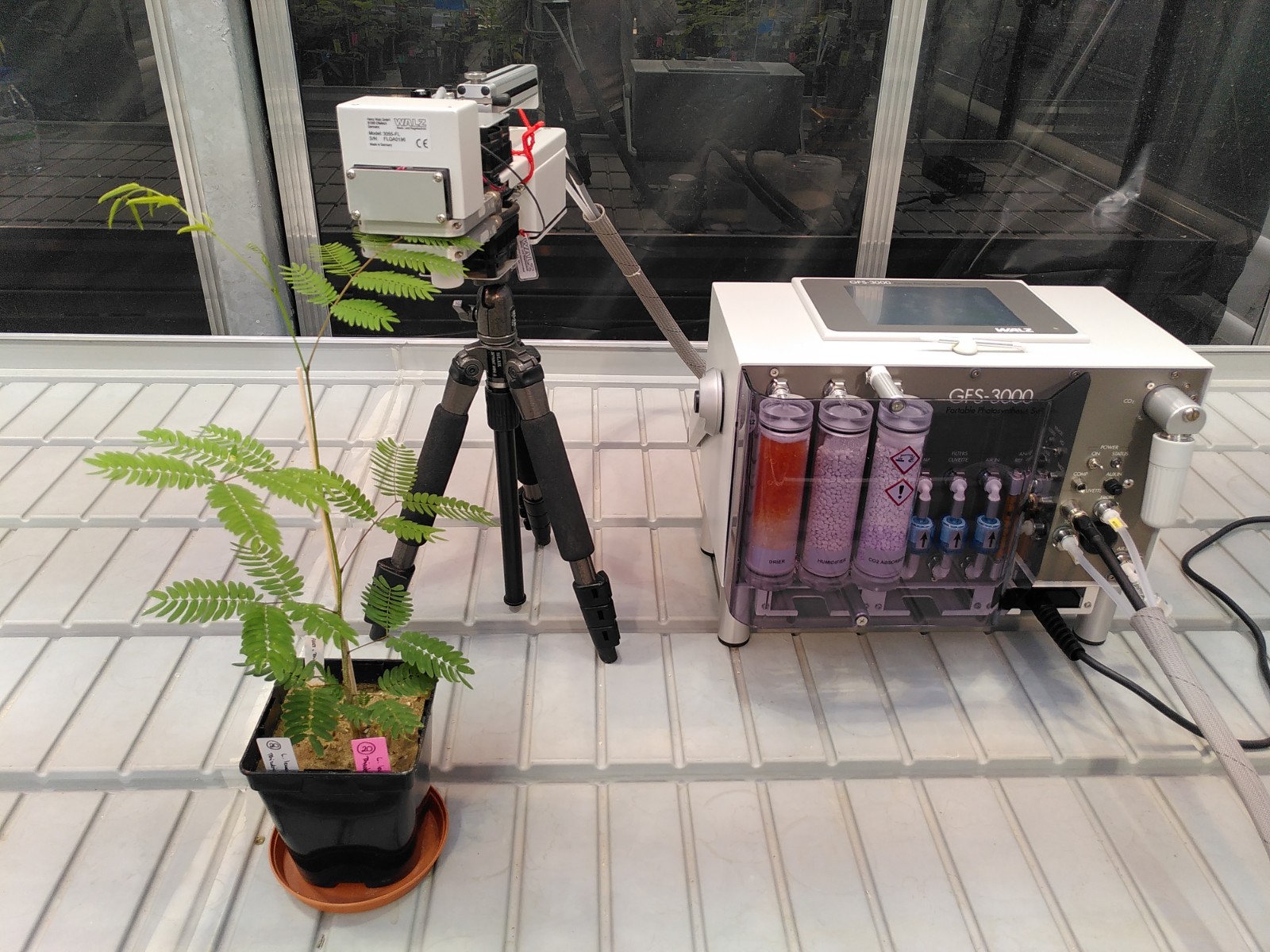

Supplement: Supplementary file 1 — (JPG 429 KB) [file 11356_2026_38009_MOESM1_ESM.jpg]

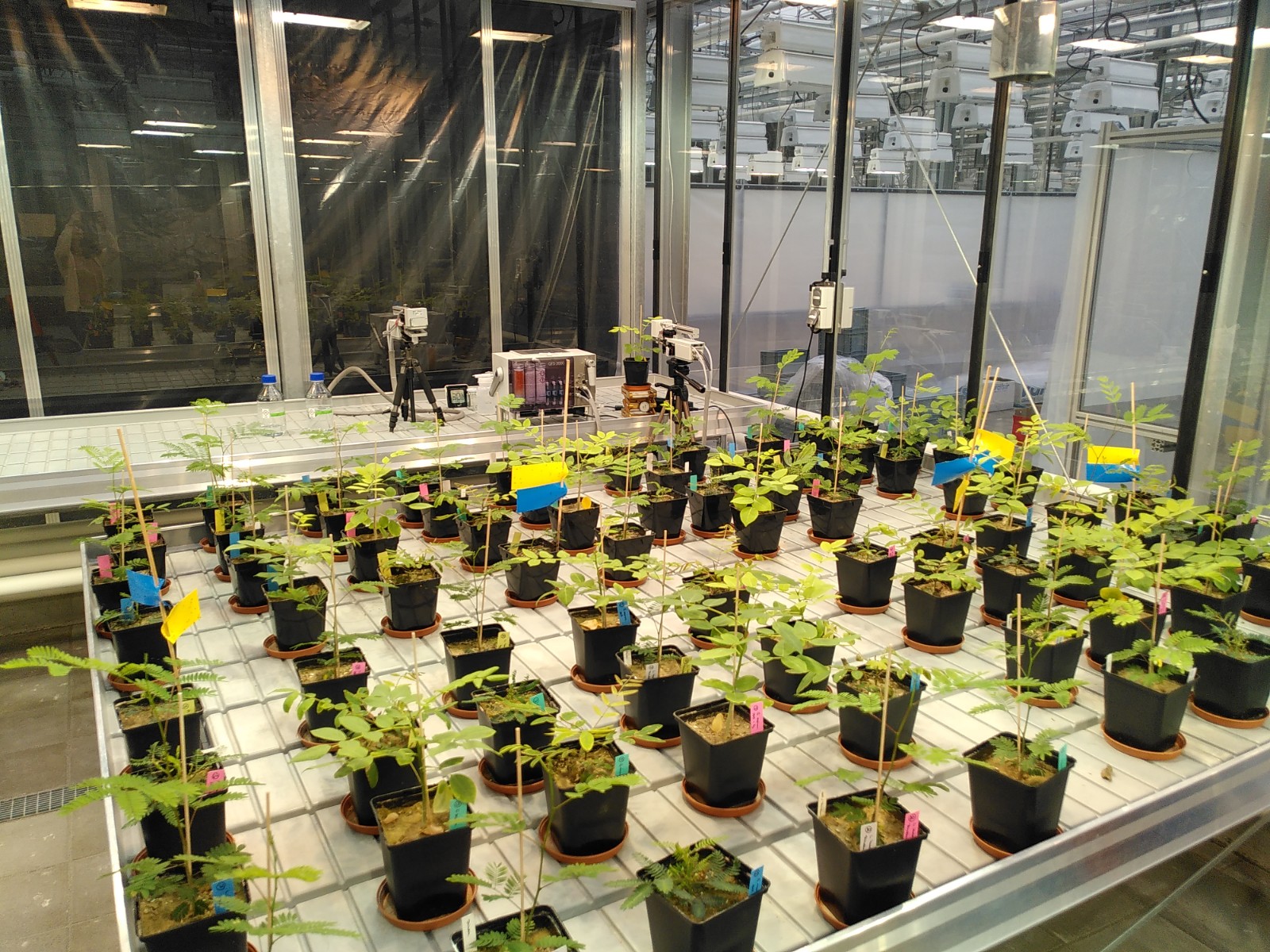

Supplement: Supplementary file 2 — (JPG 598 KB) [file 11356_2026_38009_MOESM2_ESM.jpg]

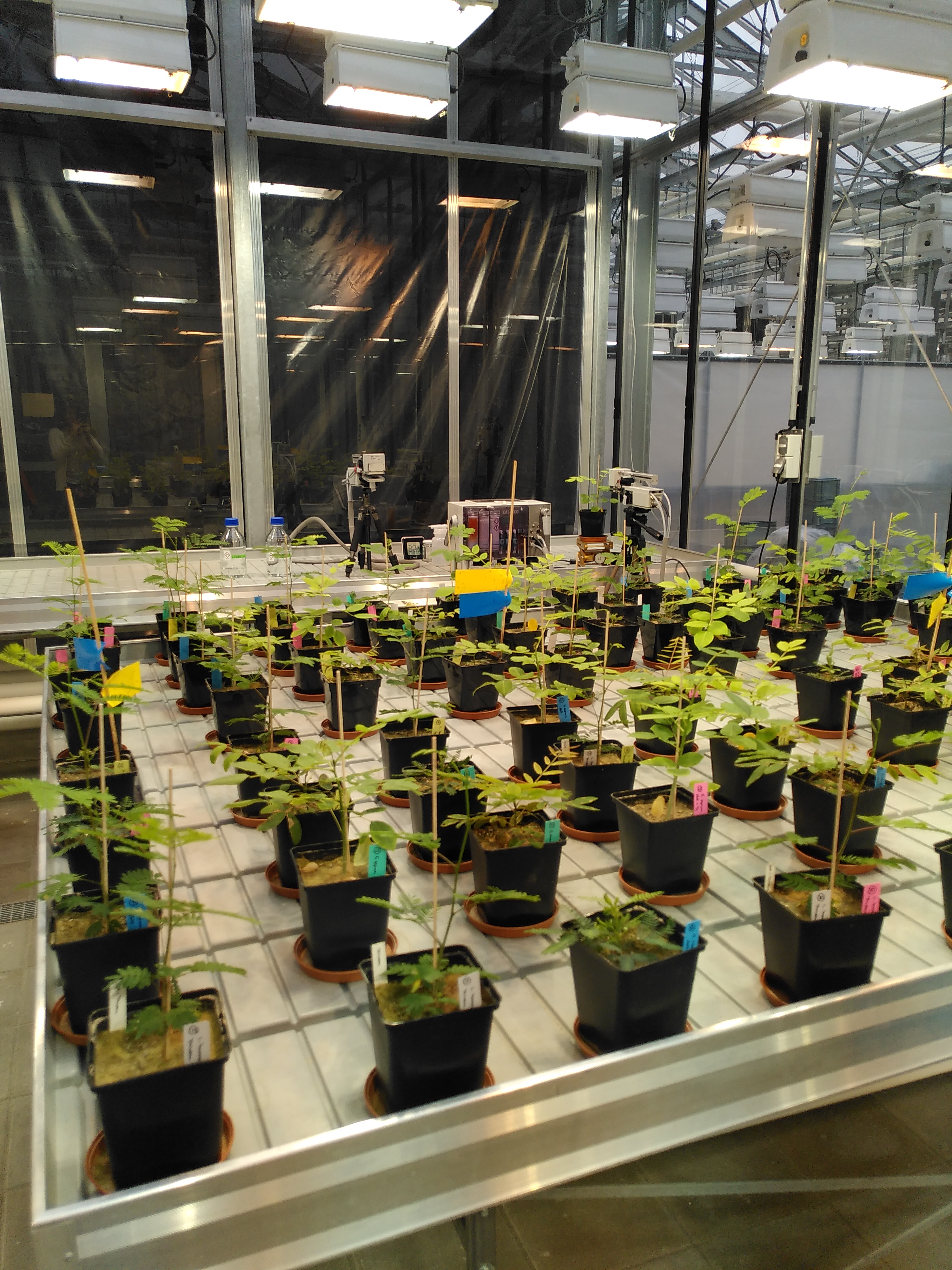

Supplement: Supplementary file 3 — (JPG 5.11 MB) [file 11356_2026_38009_MOESM3_ESM.jpg]

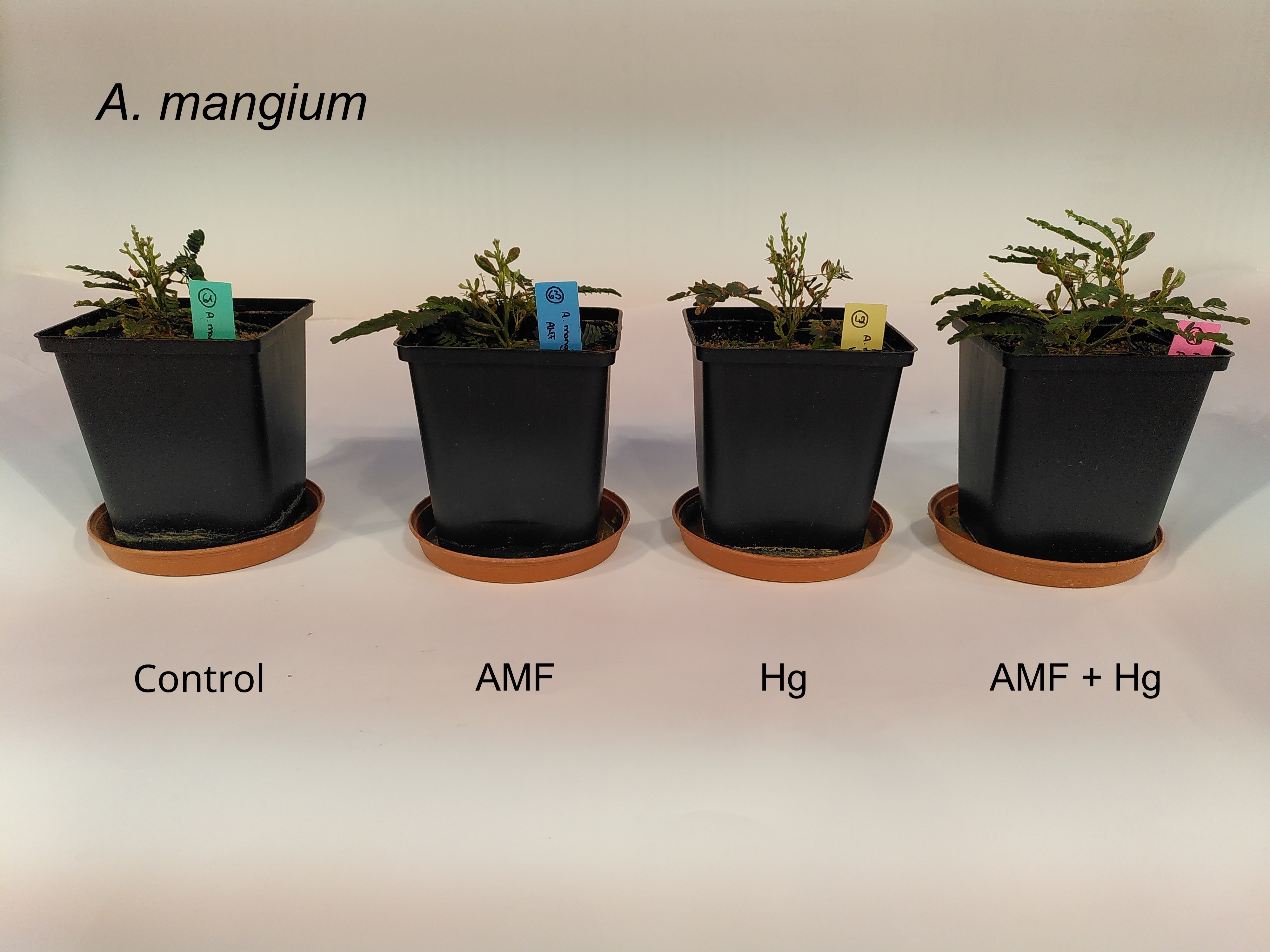

Supplement: Supplementary file 4 — (JPG 1.24 MB) [file 11356_2026_38009_MOESM4_ESM.jpg]

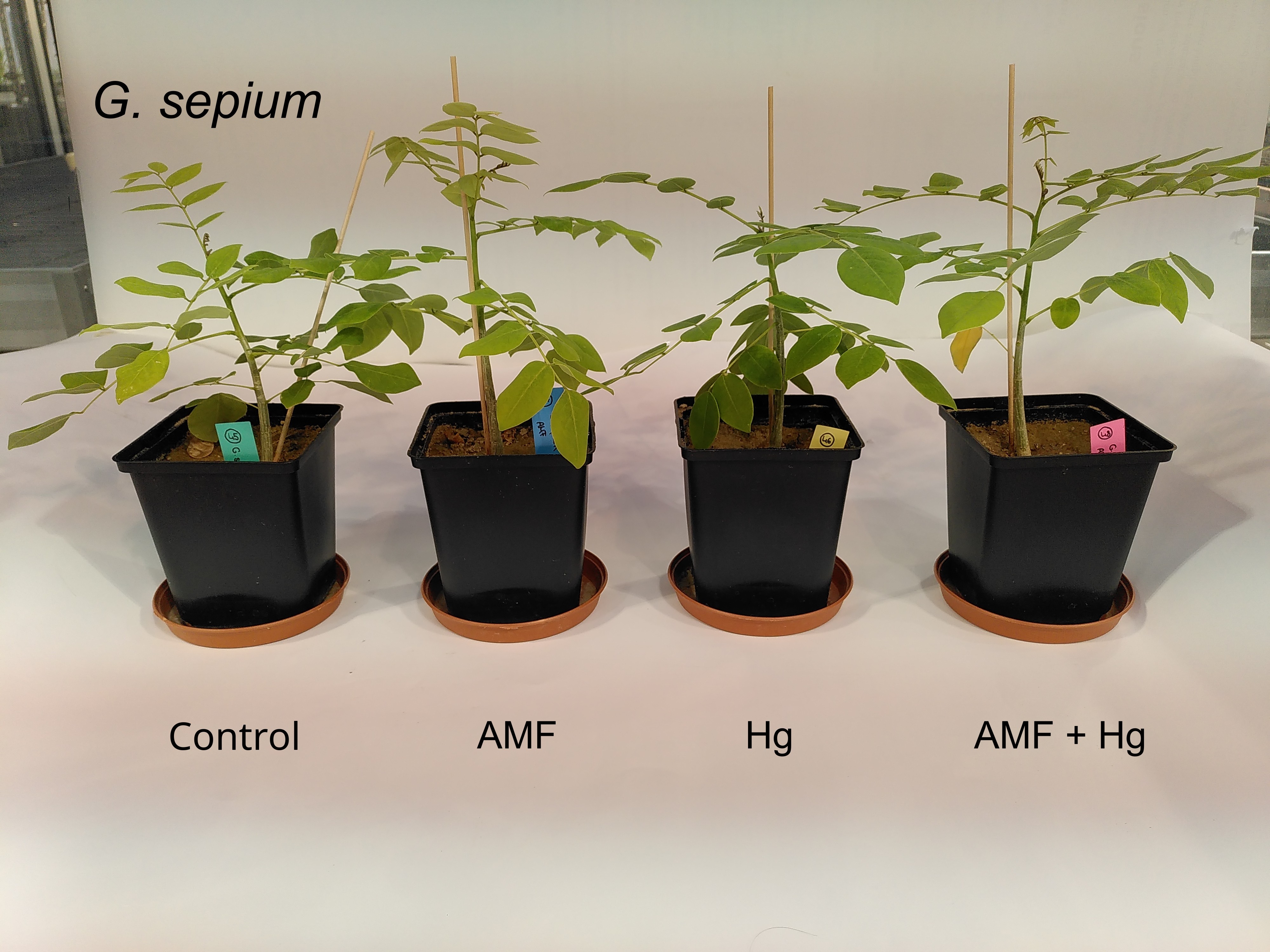

Supplement: Supplementary file 5 — (JPG 1.34 MB) [file 11356_2026_38009_MOESM5_ESM.jpg]

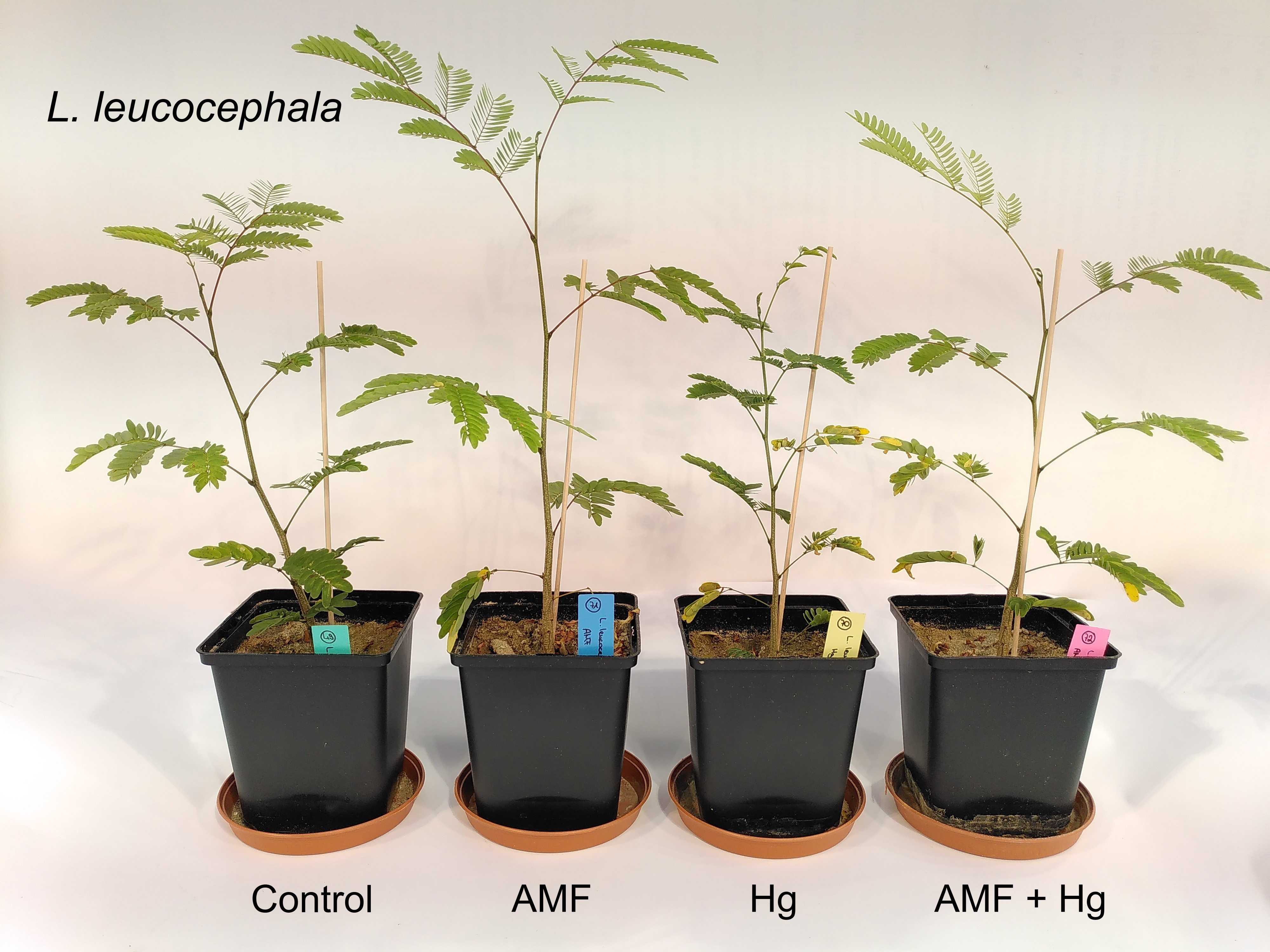

Supplement: Supplementary file 6 — (JPG 1.31 MB) [file 11356_2026_38009_MOESM6_ESM.jpg]

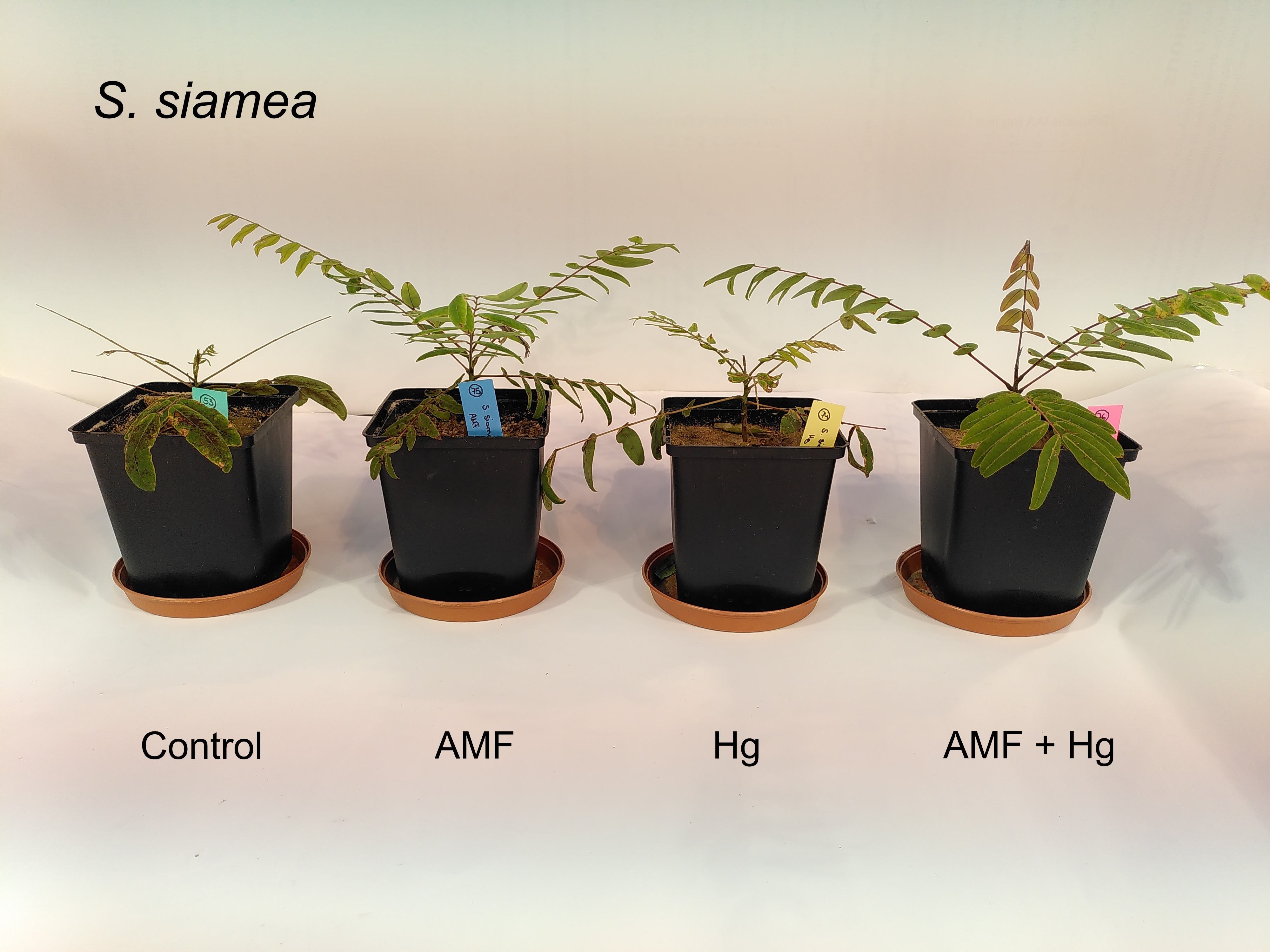

Supplement: Supplementary file 7 — (JPG 1.15 MB) [file 11356_2026_38009_MOESM7_ESM.jpg]
